# A longitudinal implementation evaluation of a physical activity program for cancer survivors: LIVESTRONG® at the YMCA

**DOI:** 10.1186/s43058-020-00051-3

**Published:** 2020-07-08

**Authors:** Jamie M. Faro, Hannah Arem, Ann-Hilary Heston, Katherine H. Hohman, Heather Hodge, Bo Wang, Stephenie C. Lemon, Thomas K. Houston, Rajani S. Sadasivam

**Affiliations:** 1 Department of Population and Quantitative Health Sciences, University of Massachusetts Medical School, 368 Plantation St, Worcester, MA 01605 USA; 2 Department of Epidemiology and Biostatistics, George Washington University, 950 New Hampshire Avenue NW, Room 514, Washington, DC 20052 USA; 3 YMCA of the USA, 101 N Upper Wacker Dr, Chicago, IL 60606 USA; 4 Wake Forest University School of Medicine, Medical Center Boulevard, Winston-Salem, NC 27157 USA

**Keywords:** Cancer, Physical activity, Implementation, Evaluation, Community-based

## Abstract

**Purpose:**

Increased physical activity (PA) levels in cancer survivors are associated with decreased risk of recurrence and mortality as well as additional positive health outcomes. PA interventions have shown to be efficacious, though many lack translation to and sustainability in community settings. We used dimensions of the RE-AIM framework to evaluate LIVESTRONG® at the YMCA, a nation-wide community-based PA program for cancer survivors delivered at Ys.

**Methods:**

This was a longitudinal study design using national LIVESTRONG at the YMCA data compiled between 2010 and 2018. Data is from all YMCAs who deliver LIVESTRONG at the YMCA, submitted by Program Directors to the YMCA-USA. We assessed reach (number of participants), adoption (associations offering the program), implementation (conducting 3 fidelity checks), and organizational level maintenance (associations recently offering program). We also examined relationships between organizational characteristics (years of program existence and association area household income) and program implementation factors with member conversion rates.

**Results:**

As of 2018, LIVESTRONG at the YMCA has reached 62,044 survivors and 245 of the 840 (29.2%) of Y associations have adopted the program. Among the adopters, 91% were aware of fidelity checks; implementation of observational (62.3%), goal setting (49.9%), and functional (64.6%) checklists varied. Most (95.1%) adopters reported offering ≥ 1 LIVESTRONG session per year (organizational-level maintenance) and a facility-level mean membership conversion percentage of 46.9 ± 31.2%. Fewer years implementing the program and higher association area household income were significantly associated with a greater membership conversion rate vs their comparison. In a multiple regression model controlling for organizational characteristics, conducting the fidelity checks independently (observational, *β* = 8.41; goal-setting, *β* = 9.70; and functional, *β* = 9.61) and collectively (*β* = 10.82; 95% CI 5.90–16.80) was positively associated with higher membership conversion rates.

**Conclusions:**

LIVESTRONG at the YMCA, in its early years, has shown promise for high reach, while adoption at more associations could be facilitated. Implementing fidelity checks along with organizational characteristics were associated with membership conversion rate. Identification of association-level strategies to increase reach, adoption, implementation, and maintenance may increase the impact of this community-based PA program.

Contributions to the literature

- LIVESTRONG at the YMCA is a nationwide physical activity program with the capacity to reach many cancer survivors, though the public health impact has yet to be examined using an implementation framework.
- Although we found the program reached many survivors, increasing adoption rates has the potential to reach a greater number of survivors. Of those reached, implementation measures and program characteristics influenced the membership conversion rate.
- This study is the first to assess implementation outcomes of a survivor community-based physical activity program and highlight the need for additional insight into implementation, organizational-level characteristics, and strategies to create more sustainable programs.

## Background

In the USA, the number of cancer survivors has increased steadily as cancer death rates have decreased [1]. There were 16.9 million survivors in 2018, a number which is expected to increase to 20.3 million by 2026 [2]. Health priorities among survivors include decreasing the risk of cancer recurrence, improving the quality of life and mental health outcomes, and general health promotion [3]. Regular physical activity (PA) has been associated with lower secondary cancer recurrence and improvements in quality of life, fatigue, fitness, body composition, mood, self-esteem, and physical function [4–6]. The American College of Sports Medicine has determined the efficacy and safety of PA for survivors [7]. American Cancer Society (ACS) guidelines recommend engaging in 150 min of moderate-to-vigorous physical activity and 2 days of strength training per week [8]. However, less than < 30% of survivors are meeting guidelines [9].

As survivorship numbers grow and PA program effectiveness data increases, the need for national dissemination of programs addressing survivor needs has become evident [10]. In 2007, the YMCA of the USA (Y-USA) partnered with LIVESTRONG® to design an evidence-based 12-week exercise intervention free of cost for cancer patients and survivors, LIVESTRONG at the YMCA [10]. In brief, trained Y instructors facilitate two weekly sessions over 12 weeks to improve participants’ aerobic fitness, muscle mass, strength flexibility and balance, and social support. The program has evidenced increases in cardiorespiratory fitness, PA levels, and quality of life [11, 12]. Since the initial pilot in 2008, a national infrastructure was created to increase its dissemination and implementation [10]. Since 2010, Y-USA has collected nationwide data about the program [13]. While a prior study reported on the reach and adoption of the program as of 2015 [10], the public health field is lacking an examination of the implementation, organizational maintenance, and membership conversion rate in this evidence-based program.

The objective of this longitudinal study was to assess implementation outcomes of LIVESTRONG at the YMCA using nationwide data collected by the Y-USA between 2010 and 2018 [13]. To conduct this analysis, we used the RE-AIM (Reach, Effectiveness, Adoption, Implementation and Maintenance) framework. RE-AIM is concerned with issues related to impact in real-world settings, incorporates both individual and organizational setting level variables, and describes the population-based impact of an intervention [14]. The membership conversion rate may be one indicator of ensuring the long-term health benefits the program provides to survivors through continued physical activity; thus, a secondary aim was to determine associations between organizational characteristics and implementation measures with this RE-AIM metric. LIVESTRONG at the YMCA is the only nationwide community-based physical activity program for survivors and has been in existence for over a decade. Thus, gaining a better understanding of this large public health program that has been adopted, implemented, and maintained in real-world settings is critical to advance the field of PA and cancer survivorship. As data in this study are provided by community workers and not researchers, we are limited in some metrics and unable to examine all RE-AIM aspects. For example, effectiveness data of participant-level outcomes was not provided in this dataset but has been shown elsewhere [12, 15].

## Methods

### Study design and participants

This was a longitudinal study design using data collected by Y-USA about the LIVESTRONG at the YMCA program between 2010 and 2018. The Y serves 22 million people in over 800 associations and 2700 individual branches across all 50 states. Y associations may consist of single or multiple branches (up to 40) within their association and operate independently of other associations. The process of becoming a LIVESTRONG at the YMCA provider has been described elsewhere [10]. In brief, YMCAs interested in becoming providers apply to the Y-USA and complete a simple readiness assessment scored by two reviewers. If the YMCA receives a score indicating they have the capacity to become a provider, they will move on to complete a 6-month on-boarding and learning process. The Y-USA is the national resource office for all Ys and exists to serve Ys. Y-USA offers technical and administrative support throughout, though Association Program Directors are responsible for marketing, administration, oversight, and funding within their association. This study was approved by the Institutional Review Board of the University of Massachusetts Medical School.

### Data sources

Data for this study included (1) routine evaluation data collected between January 2010 and June 2018 from the Association Program Directors and (2) 2010 US Census data. Y data included organizational characteristics, overall number of participants completing the program (as well as broken down by a priori membership or decision to join after the program), and awareness and implementation of three fidelity check tools separately. Reporting of all data by Program Directors is encouraged, but not required. Program data is sent to Y-USA quarterly: January 1–March 31, April 1–June 30, July 1–September 30 and October 1–December 31. 2010 U.S. Census data were used to assess the household income of each association.

### Measures

#### Reach and adoption

Reach has been identified as the absolute number, proportion, and representativeness of individuals who are willing to participate in an intervention [13]. While prior studies have examined representativeness on a small scale [12, 15], we did not have access to these data. To participate in LIVESTRONG at the Y, participants must (1) be aged 18 years of older, (2) have a previous cancer diagnosis, (3) receive medical clearance, and (4) be able to attend most sessions. Due to these criteria, we were unable to determine a denominator (number of eligible participants) to determine the proportion of individuals participating. However, using national data [16], we provide an estimate of the proportion of all possible cancer survivors (as of December 31, 2018) who have completed the program. Thus, reach was defined as one of the absolute number of participants completing the program and also an estimate of the proportion of cancer survivors completing the program. Adoption refers to the representativeness and proportion of given settings that adopt a program [13]. All 840 associations are eligible to apply to become a LIVESTRONG at the YMCA provider limited by the data provided, and adoption was conceptualized as the proportion of associations (out of all 840 associations) becoming approved LIVESTRONG at the YMCA providers. We calculated this rate as the number of approved LIVESTRONG at the YMCA association providers divided by the total number of Y associations.

#### Implementation

Implementation refers to the extent to which a program is delivered as intended [13]. While implementation incorporates numerous components, we only assess one aspect; the extent to which implementers were aware of fidelity checklists and whether or not they used them. Due to limited data, we were unable to assess other implementation aspects, such as the consistency of delivery as intended (through results of the fidelity checks) and the time and cost of the program [17]. Fidelity checklists were designed specifically for LIVESTRONG at the YMCA by the program developers and implemented from January 2017 to June 2018. Program Directors were encouraged to use three fidelity tools conducted at least annually to ensure the program was being delivered as intended (1) *Observation Assessment Tool*—used by the Program Director to observe each Instructor conducting a session, (2) *Instructor Goal Setting*—following feedback from the observation, the Instructor and Program Director worked collaboratively to identify the Instructor’s areas of strength and opportunities for improvement and document these goals, and (3) *Functional Assessments Checklist*—Program Directors observed a minimum of two Functional Assessments (baseline or 12 weeks) implemented by program Instructors. Assessing whether these checks were implemented may be one preliminary method of assessing implementation prior to examining results. Thus, implementation was conceptualized as the percentage of associations implementing fidelity checks during each reporting quarter. Program Directors reported on whether they were aware of the fidelity checklists (yes/no) and whether they completed each of the three fidelity checks (yes/no). As stated above, this assessment represents only a small section of the implementation metric and obtaining data from the actual checks themselves would provide greater insight into implementation.

#### Maintenance

Maintenance can refer to the extent to which a program can be sustained over time and may be measured at both the organizational and participant level [13]. Due to only organizational-level data available, we defined maintenance as the percentage of associations offering at least one 12-week session since the last full reporting year, 2017, divided by the total number of approved LIVESTRONG at the YMCA associations.

#### Membership conversion rate

Membership conversion rate is defined as the percentage of non-members purchasing a membership following the program cessation. Membership conversion rate (rate of non-members purchasing membership) may serve as a proxy to the lack of national-level PA data following program cessation and has been shown to predict future PA [18]. Program Directors reported the number of members and non-members who completed the program and the number of non-members who became members following the program. We divided the total number completing the program who became members by the total number of non-members completing programs to obtain this rate.

#### Organizational characteristics

Y-USA maintains the number of years the association has implemented the program along with the city and zip code of the corporate branch. We categorized the duration of program implementation into low and high, by splitting associations on the median number of years (*n* = 7) offering the program. Using U.S. Census Bureau [19], median household income data was collected for each association. The sample’s area household income was divided at the median ($47,300) to classify into high- and low-household income.

### Statistical analyses

Descriptive statistics were calculated. Independent *t* tests were used to compare membership conversion rates between organizational characteristics and implementation of fidelity checklists. Due to the variability of implementing 1, 2, and all 3 checklists, we ran all checklist models independently and cumulatively. We followed bivariate analyses with multiple linear regression analyses adjusted for organizational characteristics. These were used to determine the independent relationships between the implementation of fidelity checks and membership conversion rate. Separate models were run for all 3 fidelity variables, in addition to a 4th model run using all 3 fidelity variables. Missing data were removed from the analyses. Analyses were conducted using STATA. All statistical tests were two-tailed and considered significant at *p* < 0.05.

## Results

Descriptive statistics for organizational characteristics are shown in Table 1.

**Table 1:** Descriptive statistics for associations offering LIVESTRONG at the YMCA

RE-AIM element	Variable	Total *N*, frequency (%) or *M* ± SD
Reach	Number and percentage of survivors completing program, *N* (%)	62,044 (0.004%)
Adoption	Number and percentage of Y Associations delivering program, *N* (%)	245 (29.2%)
Implementation	Percent aware of fidelity checklists^2^	89.51%
	Percent implemented Observational checklist	62.24%
	Percent implemented Goal-setting checklist	50.19%
	Percent implemented Functional checklist	65.10%
Maintenance (organizational-level)	Percentage of associations reporting ≥ 1 sessions within last full calendar year	95.1%
*Membership conversion and organizational characteristics*		
N/A	Mean membership conversion rate per association (*M* ± SD)	46.44 ± 30.9
N/A	Number of years association implementing program	6.3 ± 2.1
N/A	Mean association area household income (*M* ± SD)	53,582.42 ± 24,522.83

^1^Partial data year

^2^Fidelity checklists represent data collected in 2017–2018

### Reach and adoption

**Fig. 1:**
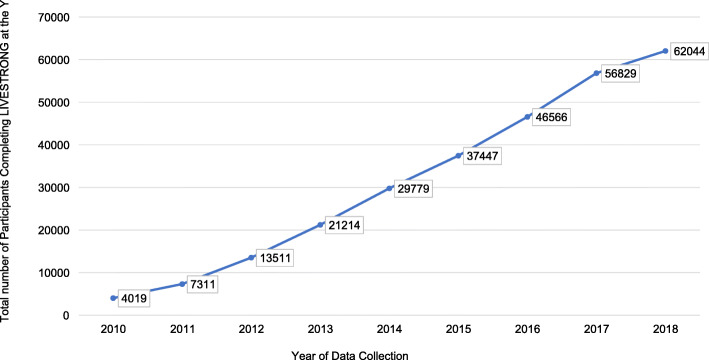
Cumulative number of participants completing the program between the years of 2010 and 2018

**Fig. 2:**
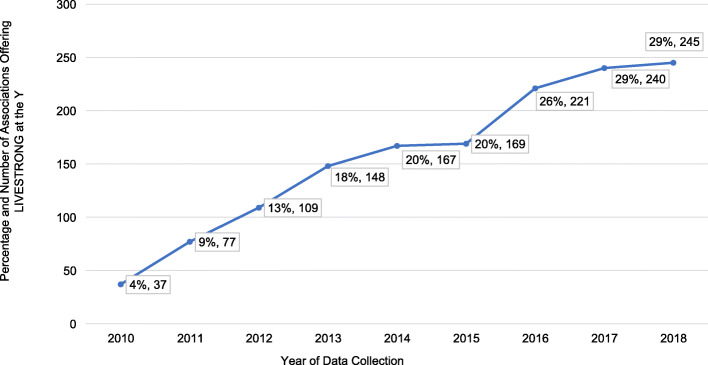
Adoption rate and number of associations trained to deliver LIVESTRONG at the YMCA between the years of 2010 and 2018

Figure 1 shows the number of participants completing the program per year. In 2010, 4019 participants had completed the program, with that number increasing to 62,044 by 2018. The number of participants completing the program has steadily increased, with more rapid increases in recent reporting years. As of December 31, 2018, there were 16,806,760 US cancer survivors > 20 years of age [16]. Thus, the proportion of survivors completing the program of all US survivors was 0.004% (Table 1). Figure 2 shows the number of associations offering the program per year. The adoption rate steadily increased apart from 2014 to 2015 and was 29.2% as of June 2018.

### Implementation

During 2017–2018, an estimated 91% (*n* = 233) of Y associations offering LIVESTRONG at the YMCA were aware of the fidelity checklists. Of those aware of checklists, 62.6% implemented the observational assessment checklist, 50.2% the goal-setting checklist, and 65.1% the functional assessment checklist. Of those associations aware of all checklists, 62.3% implemented the observational assessment checklist, 49.9% the goal-setting checklist, and 64.6% the functional assessment checklists, while only 40% (implemented all three checklists). Thus, of the associations aware of the checklists (*n* = 223), 144 did not implement all three checklists.

### Maintenance

At the organizational level, sessions were offered during 68.2% of possible reporting quarters, while 95.1% of all on-boarded associations offered at least one session since the reporting year 2017.

### Factors associated with membership conversion rates

The mean membership conversion rate was 46.4% ± 30.9, with a range from 0 to 100%. Membership conversion rates were significantly greater in associations that implemented observational, goal-setting, and functional checklists independently (see Table 2). We found similar results for those associations implementing all three checklists. In a linear regression model adjusting for organizational characteristics, implementing observational (*β* = 8.40, 95% CI 3.33–14.67), goal-setting (*β* = 9.7, 95% CI 4.85–16.63), and functional (*β* = 9.61, 95% CI 3.98–15.6) checklists independently and collectively (*β* = 10.82; 95% CI 5.90–16.80) were significantly associated with greater membership conversion rates. Associations implementing the program for < 7 years had a significantly greater membership conversion rate than those implementing for ≥ 7 years. The membership conversion rate was significantly greater in higher household income areas as compared to lower household income areas (48.5 vs. 44.29; see Table 2).

**Table 2:** Independent sample *t* tests comparing membership conversion rates between organizational characteristics and fidelity checklist awareness and implementation

Variable	*Membership conversion rate (M ± SD)*	*95% CI*	*p value*
*Organizational characteristics*			
Time implementing program (years)			< 0.001
< 7 years	49.3 ± 31.6	47.6 to 51.0	
> 7 or more years	42.8 ± 29.7	41.0 to 44.6	
Association area household income (median)*			0.001
≤ 47,300	44.3 ± 30.3	42.5 to 46.1	
> $47, 300	48.5 ± 31.4	46.7 to 50.2	
Fidelity checklists**			
Aware of fidelity checklists			0.832
Yes	46.6 ± 37.5	37.3 to 56.0	
No	47.5 ± 32.1	44.8 to 50.3	
Implemented observational checklist			0.002
Yes	50.6 ± 1.7	47.2 to 54.0	
No	41.6 ± 30.3	37.2 to 46.0	
Implemented goal setting checklist			< 0.001
Yes	52.5 ± 1.9	48.7 to 56.3	
No	42.2 ± 30.7	38.4 to 46.0	
Implemented functional checklist			0.001
Yes	50.6 ± 32.4	47.2 to 54.0	
No	40.8 ± 30.4	36.2 to 45.4	
Implemented all 3 checklists			< 0.001
Yes	54.2 ± 32.3	49.9 to 58.4	
No	42.8 ± 31.2	39.3 to 46.3	

*P* value significant at < 0.05

*Association area median household income was acquired using data from the US Census Bureau

**Fidelity checks only apply to data collected in 2017–2018

## Discussion

Our results suggest that while LIVESTRONG at the YMCA has linearly increased its participant reach and organizational adoption rates, it has room to continue to grow. Implementing community-based programs with high fidelity is challenging, and our findings suggest implementation measures of fidelity checks can be improved. While the data only shows whether checks were implemented and not actual fidelity to the program, we did find implementing checks were associated with membership conversion rates. Further, we found that area household income was associated with membership conversion rates as well. This supports the notion that inequities exist in survivor PA and potentially access to community-based programs, in low socioeconomic status areas [20].

The estimated 60,000 survivors that the program has reached is only a small fraction of the roughly 16.9 million survivors in 2018 [2]. In an attempt to provide a proportion for the number of survivors participating in the program, we found that 0.004% of US survivors completed the program. However, this value does not consider medical eligibility, location to the nearest YMCA, or other contextual factors needed to determine eligible participants. Increasing the number of participants is an important research priority. A prior examination of a subset of program participants (7%) showed the highest reported method of referral to LIVESTRONG at the YMCA was from a doctor or other healthcare professional [10]. Providers may serve on the front line to screen and refer patients to appropriate programs that fit their medical, geographic, social, and economic preferences [21]. Recent health reforms have placed an emphasis on using electronic medical health records for surveillance [22]. Thus, integrating PA surveillance into the standard of care may provide better insight into patient characteristics, medical clearances, and referral to appropriate PA programs, such as LIVESTRONG at the YMCA.

Reach may also be increased if additional Ys adopt the program, though strategies are still needed. A previous study examining a health program in Y-affiliated sites found that adoption facilitators included organizational support, on-going financial support, matching the Ys mission and target population, novelty of the program, invitations from established partners, and program champions [23]. Barriers included limited resources and expertise, competing programs, and space and costs of offering the program. A prior examination of the Diabetes Prevention Program delivered in YMCAs found that outreach and recruitment required 2 to 20 h of staff time per week [24]. As LIVESTRONG at the YMCA is free of cost, the YMCA staff must use some of their time to employ fundraising efforts to fund the program. Alleviating staff time and the financial burden of program costs may increase the adoption of the program and staff time to devote to outreach. The American Society of Clinical Oncology has encouraged a third-party payer system to provide coverage of services for cancer prevention and control, including those for PA [25]. Payer financial assistance may alleviate fundraising burden from Ys and provide opportunities for more Ys to adopt the program, run additional sessions, perform outreach efforts, and reach more individuals.

Program fidelity may potentially moderate the relationship between an intervention and its outcomes [26]. Less than 40% of associations were implementing all three checks which were associated with greater membership conversion rates. Fidelity is associated with an intervention’s outcomes [27], and incorporating checklists is one way to measure adherence to delivering the intervention as intended. However, fidelity monitoring delivered in non-research-based settings presents several logistical concerns of self-report measures, time, and resources to complete checks while concurrently implementing the program, as well adaption to the local setting and drift from the intervention [28]. Implementation strategies may be needed to promote fidelity. We also found that fewer years implementing the program was associated with a higher membership conversion rate. Examining setting-specific variables affecting programs implemented over a longer time, such as funding, community saturation, change in organizational structure, adaptability of the intervention, and support from leadership [29], are warranted.

Consistent with prior data that the purchase of a fitness membership is limited to those of higher socioeconomic status [30, 31], we found that household area income was associated with membership conversion rate despite that the Y offers financial assistance to those in need. Strategies to motivate and support participants facing financial stress are needed to reduce the disparities in participation. Survivors have reported financial constraints as a barrier to exercise [32]. They also report spending 1/3 of their household income on cancer care [33]. Third-party payer systems covering survivor PA services may provide a re-allocation of funds to overcome financial barriers to program attendance, including childcare, transportation, and athletic gear. Additionally, there are considerable disparities in the population being served in PA programs for survivors [34]; thus, there is a need to determine how to make even free-of-cost programs more accessible to minority survivors and those with low socioeconomic status. Providers may be able to assist in these efforts, as ACS guidelines and the Institute of Medicine recommend PA prescriptions and/or referrals be provided to survivors. However, specific recommendations on how to prescribe or where to refer patients are not included [35]. Provider education coupled with assessing barriers to PA may aid in the PA referral process.

Several limitations should be noted. First, data is optionally self-reported from Program Directors; thus, it is unclear if an association with no report conducted sessions and our results may underestimate the outcomes of interest. Second, data is reported from Y associations rather than individual branches; therefore, it is unclear as to how individual branches perform within each association as well as a lack of branch-specific contextual factors (such as staffing, financial resources, facilities, equipment, and leadership) which may influence the capacity and performance of programs. Third, the RE-AIM metrics identified in this study are limited to the data provided. This is a strength, as measures are collected by all associations similarly, though a weakness as these measures do not fully capture all indicators of each RE-AIM aspect (such as the unknown characteristics of those not participating in the program, a denominator that captures all eligible participants for reach and the number of associations who applied to become a LIVESTRONG provider but were not approved or failed to complete the training). In lieu of long-term PA maintenance measures, our data was limited to membership conversion rates upon program cessation, which is not a construct within the RE-AIM framework. This measure also does not account for those who purchase a membership later nor assess membership use or PA behaviors in alternative settings. Fourth, our metric of household income based on census data is limited to the corporate branch within the association. Not having data at the level of the implementing branches limited our ability to understand the implementation context. Lastly, the metric of associations offering at least one session per full reporting year provides only preliminary insight into an association’s organizational maintenance.

## Conclusions

Applying RE-AIM to evaluate a community-based health program presents a number of complexities that are not present in traditional research-based programs [36]. We provided an examination into the implementation of this program, which will become more needed as the number of cancer survivors increases and opportunities for structured, evidence-based PA programs become critical. LIVESTRONG at the YMCA has the potential to reach many communities, successfully implement, sustain, and expand the program over the course of a decade. However, disparities in the programs’ reach remain, and processes need to be integrated into the standard of care to screen and refer survivors. Future efforts should address setting-specific contextual factors to allow for the identification of strategies and tools to enhance program implementation and maintenance. These efforts will be strengthened by studies that assess all RE-AIM measures.

## Data Availability

The data that support the findings of this study are available from Y-USA but restrictions apply to the availability of these data, which were used under license for the current study, and so are not publicly available. Data are however available from the authors upon reasonable request and with permission of Y-USA.

## Abbreviations

ACS: American Cancer Society
PA: Physical activity
RE-AIM: Reach Effectiveness Adoption Implementation Maintenance
YMCA: Young Men’s Christian Association
